# Digital loneliness—changes of social recognition through AI companions

**DOI:** 10.3389/fdgth.2024.1281037

**Published:** 2024-03-05

**Authors:** Kerrin Artemis Jacobs

**Affiliations:** ^1^ Department of Philosophy, Ethics, and Religious Studies, Faculty of Humanities and Human Sciences (Graduate School), University of Hokkaido, Sapporo, Japan; ^2^ Center for Human Nature, Artificial Intelligence, and Neuroscience (CHAIN), University of Hokkaido, Sapporo, Japan

**Keywords:** (digital) loneliness, recognition, AI companionship, critical theory, meaningful relations

## Abstract

Inherent to the experience of loneliness is a significant change of meaningful relatedness that (usually negatively) affects a person's relationship to self and others. This paper goes beyond a purely subjective-phenomenological description of individual suffering by emphasizing loneliness as a symptomatic expression of distortions of social recognition relations. Where there is loneliness, a recognition relation has changed. Most societies face an increase in loneliness among all groups of their population, and this sheds light on the reproduction conditions of social integration and inclusion. These functions are essential lifeworldly components of social cohesion and wellbeing. This study asks whether “social” AI promotes these societal success goals of social integration of lonely people. The increasing tendency to regard AI Companions (*AICs*) as reproducers of adequate recognition is critically discussed with this review. My skepticism requires further justification, especially as a large portion of sociopolitical prevention efforts aim to fight an increase of loneliness primarily with digital strategies. I will argue that *AIC*s rather reproduce than sustainably reduce the pathodynamics of loneliness: loneliness gets simply “digitized.”

## 1 Introduction: the digital turn of social recognition

It is evident that a *digitalization of human relations* is affecting our social recognition relations, particularly with respect to the conditions of social integration and participation. What on the one hand may lead to stronger networking, the possibility of faster exchange of information and social visibility, and empowerment, comes with the social challenges posed by loneliness as a *social pathology* (1, 2). The sociopathological dimension of loneliness becomes visible not only in the (inter-)subjective phenomenality of suffering but is also assessed against the backdrop of systemically induced changes of the lifeworld, such as the use of highly advanced social AI. For highly digitized societies—think for instance of Japan, whose industry invests millions in the production of *x-bots*^1^ to compensate for the shortage of skilled nursing staff^2^—AI Companions (*AIC*s) are apparently perceived as an adequate technical (systemic) strategy for dealing with societal problems such as endemic loneliness anticipated in the future. In an ideal scenario, social AI would contribute to a change of systemic and lifeworldly structures in the direction of the enhanced social integration of lonely people, and future loneliness prevention. This makes it necessary to critically assess the effects of digital solutions for global “loneliness management,” particularly against the backdrop of current medical and epidemiological research: Persistent experiences of loneliness (3) are considered alongside other well-known factors such as poor nutrition, stress, noise, or low socioeconomic status (4) for their pathogenic potential [cf. (5–8)] so that effective loneliness prevention would significantly change the onset, manifestation, and persistence of specific illnesses [e.g., depression; cf. (9–11)] in recent societies. Moreover, a sheer *quantity* of social relationships and optimal networks between people nevertheless apparently cannot prevent people from feeling lonely, isolated, and socially excluded (12). This correlates with individual disposition and resilience factors, e.g., with certain personality *traits* (8, 13) as empirical studies have shown (14), and depends on the personal attitude according to which someone evaluates one's own situation *as* loneliness. When *non-trivial*^3^ suffering from loneliness occurs, i.e., when loneliness is not only recognized as a potential threat to individual health alone, but also seen for the particular *social miseries* that it produces, it can be reconsidered as a new form of *precariat*. Apparently particularly vulnerable social groups [e.g., the ill, the elderly, and the socioeconomically disadvantaged, particularly children (15–17) and young adults (18)], are especially affected by it. This requires interdisciplinary societal (e.g., healthcare, political, medical, ethical, etc.) intervention strategies^4^.

Many interventionist strategies for reducing social isolation (19) have been formulated over the years. Loneliness prevention is a top goal of public health and “built environment” projects (20), while it has just recently become a major topic of global healthcare politics and governmental action (21, 22). What current interventionist approaches could stress more is that loneliness is a symptom of disturbed social recognition relations. I will explain this in a first step in specifying *digital loneliness* as implying significant alterations of *meaningful relatedness* (23–25). Although there are also some positive, affirmative readings of loneliness—for instance, its praise as *solitude* (26, 27)^5^—this paper is concerned rather with its shadow side. Problematic changes of personal relationships in the digital age have been already outlined with respect to the alienation phenomena [e.g., in terms of “self-commodification” (29), “acceleration” (30), and “atomization” (31)] under the auspices of a cultural-reflexive analysis.

This allows one to specify loneliness as a painful experience of a lack of or false social recognition in the context of a (cultural) theory of the digital. My question is whether AIC might be able to compensate for and even provide some effective forms of social recognition to prevent such experiences of alienation. One can be concerned with the increasing tendency to regard AICs as reproducers of adequate recognition. Despite all the fascination with *AIC*s, this philosophical-critical review asks whether these are changing a certain understanding of social recognition in a lasting, maybe not solely positive way. The *digitalization of detachment* for which I believe human–*AIC* relations paradigmatically stand can be highlighted: What appears surmountable with an *AIC* as a *relational artifact* (32) is actually often reproduced by it: digital loneliness! A critical theoretical view on AI companionship respectively must ask for the normative consequences of treating social AI *as if* it were human, which is sketched in a second step. With a view on the embedding conditions of social AI, there are reasons to claim that one probably *shouldn’t* spend too much time with a robot companion. This opposes the narrative of *AIC*s as adequate substitutes for human forms of relatedness. It is concluded in a third step that the social malaise of epidemic loneliness is the result of attempts to solve non-trivial suffering with AI cognition rather than human recognition. This may be considered ethically misguided if the practice of humanizing AI comes at the expense of a dehumanization of relatedness, which is the conclusion of this analysis.

## 2 (Digital) loneliness—connected, yet alone!

There are many theories, and multifold interdisciplinary readings, of the notion of loneliness. Weiss (33) has stressed a methodological flaw of loneliness research as follows:

What seems to me wrong with many current “definitions” of loneliness is that they are insufficiently sensitive to loneliness's status as a real phenomenon. (…) They define it by the conditions that might theoretically give rise to it. (…) Other definitions of loneliness suggest a theoretical idea of what is at the heart of loneliness. (…) Actually, these not only are not descriptions, they are not definitions. They are mini-theories. By wrapping together identification of the phenomenon (“*this* is loneliness”) with an explanation for the phenomenon, they foreclose the critical research question (33).

To avoid literally foreclosing the *critical* research question, it might be helpful to ask what can be derived *from different disciplinary perspectives* on loneliness for my reconceptualizing of loneliness as a symptom of distorted social recognition relations. I have suggested an understanding of “loneliness” as a significant change of meaningful relatedness against the backdrop of three anthropological premises: (1) we are (like other animals) relational beings, (2) as such, we need some sort of social recognition (predominantly in the basic forms of mutual empathic understanding and respect), and (3) we can suffer from loneliness, because we are sentient beings [cf. (25, 34)]. This makes it plausible why we often evaluate loneliness as a condition that is impairing or negatively interfering with our wellbeing [see also (35)], and our health respectively, as the notion of wellbeing is essential to an understanding of psychosocial health (36–39). Loneliness affects the wholeness of one's self-world relation: it changes the evaluative processes of a person (i.e., its cognitive, affective, volitional patterns of enaction), and therefore changes the way in which someone relates to self, others, and the world: it apparently always takes place in social embedding relationships, i.e., there is no such thing as loneliness without the (embedding) sphere of the social. One can be (physically) alone without feeling lonely, and, moreover, one can be around others, and yet, still feel fundamentally lonely (40, 41). It is apparently the *quality of relatedness* to others that changes: Loneliness occurs because a relationship does not attain an expected or desired level of quality and significance (42). Thus, it strikingly reveals itself when one is around others. This points to the important difference, but also relation between “external” factors (like physical isolation or being socially excluded from a group), and the inner corresponding feelings or stances regarding these experiences (43, 44). The peculiar double-aspectivity of loneliness as a contact-psychological ambivalent situation is captured in reminding that it is often experienced both as a burden *and* as a requirement, e.g., it can be of some sort of instrumental value, for instance for personal goals of contemplation or recovery (45). If we place loneliness within the differentiated structure of *interpersonal relatedness* (46), it acquires its meaning only through the reverberation of past relationships or the anticipation of future ones. What hurts in loneliness is particularly the loss of emotional and intellectual closeness to others, which often reveals experiences of being “socially invisible,” excluded, not appreciated, neglected, being an “outcast,” etc. (47). Loneliness therefore can be called a social pathology because it is an expression of “*dysfunctions that violate a society as a whole at the sensitive interface of individuation and social integration*” (48). It is a phenomenon that is caused by disturbed symbolic reproduction dynamics of the lifeworld. These become more specifically graspable as the result of failed processes of social integration. Attempts to cure this with *AIC*s might exemplify what “goes wrong” in these digital processes of integration. The philosopher Axel Honneth has described these pathogen dynamics as a “forgetfulness” of social recognition, according to which one can also differentiate forms of *social invisibility* (49), respective strategies of the “invisibilization” of others, which is one way to explain the dynamics of social disintegration and marginalization of individuals and groups. In principle, the adoption of an objectifying stance can be normatively permissible in many cases, but with his analysis of a “forgetfulness of recognition,” one can focus on the dynamics of reification that often erodes the very preconditions for any trustful intersubjective practice and corrupts basic modes of mutual respect and understanding, thus the prerequisites for ethical practice in the social sphere. Honneth says “this kind of ‘forgetfulness of recognition’ can now be termed ‘reification.’ I thereby mean to indicate the process by which we lose the consciousness of the degree to which we owe our knowledge and cognition of other persons to an antecedent stance of empathetic engagement and recognition” [cf. (50)]. We *suffer* from loneliness because we perceive instantly what is essentially missing: a relatedness to others in which we experience ourselves as adequately socially recognized. The crucial point of Honneth's theory—therein adding something new to the standard view on loneliness as mere subjective feeling (51)—is that for many loners this goes hand in hand with the suffering from being ostracized, rejected, overlooked, not taken seriously as a participant (e.g., by their families, peers, at the workplace, by authorities), which places loneliness within a broader frame of the reproduction dynamics of social recognition: One must therefore discriminate between either a *lack* of or expressions of *false* recognition, which both include (sometimes: intentional) strategies of “invisibilization”: While a *lack* of social recognition implies a fundamental *neglect* of the other [an can also include the intention to harm someone through neglect, e.g., by objectifying someone as a *mere enemy*, as Sticker (52) has described it], *false social recognition* refers to the instrumentalization of agents by others, i.e., being valued as a *mere means* to an end for the other, which equally can cut people off from basic social inclusion. This lack of empathy is a “deficiency” mode of social recognition. Interactions that cannot reproduce the respective forms of social inclusiveness and integration can be assessed not only as potentially unethical and/or legally problematic, but also as contributing to a perpetuation of a social malpractice that alienates people and defines the status of loneliness as a socially precarious condition. At the moments when people “cannot conceive of themselves as actively participating and interrelated members of a jointly experienceable society” (48), the (pathogenic) potential of loneliness as both a cause and symptomatically an expression of disturbed recognition relations can be stressed. This negative experience can induce agents to socially withdraw from such social constellations to seek out more satisfying connections, and this, in principle, does not rule out the fact that basic experiences of appreciation are sought and (allegedly) found in relation to social AI. It seems uncontroversial to claim that *AIC*s do “re*cognize*” humans in a technical sense, but my point is to ask whether we can really assume capabilities for *re*cognition. If it is reasonable to accept that loneliness is the kind of alienation experience that evolves out of and is an expression of distorted social recognition relations (graspable as a lack of, or a false recognition practice), and social AI is designed to fill precisely this gap of an experienced lack of relatedness or should even compensate for experiences of false recognition, it is fair enough to question whether *AICs*, no matter how “sociable” they appear, can really help to overcome loneliness. This would imply that lonely people are placed in a relation that basically allows them to feel connected in the first place. While I believe that *AIC*s can provide this basic feeling of “connectedness,” one can doubt whether these devices sufficiently contribute to the kind of social inclusion that people experiencing loneliness really need. I suspect that we are rather dealing with the digital variant of a fundamental lack of recognition in human–AI companionship, albeit with *AIC*s delivering the perfect illusion of recognition. Ergo there might be the concern that *AIC*s contribute to sustaining loneliness rather than offering a way out of it. But how do the (patho) dynamics of loneliness relate to altered social recognition?

### 2.1 Human companionship: recognition first!

If “relatedness” is the backdrop for perceiving loneliness as a state in which something is fundamentally “missing,” the *inherently evaluative endeavor* that is crucial for all dynamics of *intersubjective* encounters (53, 54) becomes relevant. People are involved in all kinds of *interactivity* that often gives rise to the particular experience of a “*we*-feeling” (55, 56) or accompanies experiences of a *being-with* (57). In the suggested reading here of the notion of loneliness it is not stressed as a “synthetic *a priori*” of human consciousness [as, e.g., suggested by Mijuskovic (58); for review, see Jacobs (59)], but rather is perceived as a condition that necessarily has to be commemorative of the *primordiality* of social recognition (50, 60). This primordiality of (affective) relatedness is the ontological and conceptual prerequisite for being able to experience any “lack” of it. This may become more plausible when we stress the developmental aspect of social cognition (61–63). Through the recognitional “interactive” modes of imitation, joint attention, as well as affective contagion, we can presuppose a *priority of re*cognition over cognition from a biopsychosocial developmental perspective (24, 64). Consequently, the perception of a lack of social relatedness or suffering from the pain of social disconnectedness would not even be possible without the all-important (basically affective) experiences of previous interactions that impregnate our brains (61). Thus, what some epistemological approaches to loneliness á la Mijuskovic mostly fail to address is these core experiences of intersubjectivity. These are the experiential prerequisites for being able to register an impairment of relational experience. This often is accompanied by an additional feeling or judgment, e.g., that it is (felt as) unpleasant, distressing, impairing. Consistent with this view is that experiences of loneliness play a necessary role in the individuation process, as it has been exemplarily outlined by Winnicott (65) and in other theories that focus on social relatedness and its disorders, respectively (66–70). Although loneliness clearly has an affective dimension (we often *feel* lonely, when *we are* lonely), it nevertheless is not a distinct emotion. Rather, it is the framing condition for very different emotional episodes to appear (e.g., fear, sadness, forlornness, etc.), and, simultaneously, reveals our affective vulnerability: the suffering from loneliness literally can be nerve-wracking, as it is, after all, associated with a state of *emotional distress*, which occurs as a reaction to experiences of being alienated or misunderstood, socially rejected, and/or otherwise restricted in opportunities for emotional intimacy with others [cf. (71)]. Neurobiological research moreover associates the processing of experiences of social exclusion in the *Anterior Cingulate Cortex* where physical pain is processed [cf. (72–74)]. This “social pain” hypothesis of loneliness can be further linked to the evolutionary view on its supposable (mal-)adaptive functions. Here it seems that feeling lonely motivates people to seek contact with others (75). By contrast, its maladaptive effects have been discussed along the lines of the abovementioned inherent social exclusion dynamics: It is true that loners often fight a “*struggle for social recognition*,” i.e., that being lonely implies unequal treatment or a lack of social participation that negatively affects the self-relation of a person (76). It has been shown that loneliness is associated with stigmatization processes (77), experiences of shame (78), and often leads to situation in which loners are intentionally socially shunned *because of the very fact that* they are lonely [cf. (79)]. This social exclusion of lonely people has been explained with studies on emotional contagion that provide evidence that loneliness “spreads” (80) among even larger populations like a virus. For vulnerable people, this often comes with the experience of social rejection and isolation as their isolation is (sub-) consciously perceived by others who do not offer support to loners but display quite the opposite behavior to “protect” themselves from “catching” it. It is these inherent social dynamics of loneliness as an increasing process (germ. *Vereinsamung*) by which it manifests as a chronic condition. In chronic loneliness, people are no longer able to assess their condition concerning (rememberable) experiences of closeness, affective resonance, existential security, and closeness to others. This can include a reduced capability to interpret social cues correctly, i.e., it is not solely because they are lonely that people are shunned, but also because they might display altered recognition of others, for instance, due to their anticipation of being rejected (81). Others may distance not only because they may feel overwhelmed by the lonely person's need for recognition, but because they also display socially avoidant behavior, which has been suggested as serving a self-protective function in loneliness (82).

With such an emphasis on the role of intersubjective, particularly inter-*affective* dynamics, it may appear even more plausible that sentient, relational beings are also affectively responsive toward objects like *AICs,* even developing a bond to these devices [cf. (83)]. This seems even more likely when *x-bots* mimic human interactional patterns, which is possible as most *AIC*s come equipped, for instance, with emotion detection that allows to track and to directly “adjust” to the particular moods of people (84–86) [for a review, see Spezialetti et al. (87)]. In addition, *AIC*s are often *perceived* as objects of patience (88) and are treated not as mere “technical devices.” Instead, there is the tendency to perceive them *as if* they were human, which correlates to the extent *AIC*s appear as human-like (89). It is the relational design of adaptivity to the specific needs of human intersubjective (e.g., communicative) practice that, together with a particular responsivity of *AIC*s, can make people forget that “the subject they have called is (still!) not available.”

That being said, one can now focus on the central question: Can *AIC*s be helpful with loneliness? It seems wise to opt for a pragmatic view: one can stress the benefits and individual experience of feeling less lonely with an *AIC*, which then can be reassessed against the backdrop of possible negative impacts that this relationship (in the long run) might imply. This, however, does not rule out a more conceptual view: One might aim to answer the question of whether social AI can be adequate or sufficient for supporting people and/or for societal loneliness prevention by focusing on *features of AICs* that may allow us to assess *AIC*s as either “capable of social recognition” (which respectively also opens up the possibility for *false recognition* when being related to an *AIC*) or not capable at all by definition. Whether or not this would imply that we need an extended theory of social recognition, which must include *AIC*s as “artificial agents who are able to care,” is an additional question that may emerge from such an investigation.

### 2.2 AI companion: the subject you call is (still) not available!

The *AIC* is in a literal sense a *cultural machine*, i.e., it is itself a signature of productive and recombining cultural dynamics and is part of the complex social dynamics of digitality that has led to a singularization of the human subject (90). The *AIC* as *technical other*—unlike a coffeemaker or vacuum cleaner—apparently triggers our affective involvement. This has been evidenced, for instance, in the case of chatbot-use (91–94). It apparently matters most how *we* perceive our digital companions: it is *we* who imbue our relations to *AIC*s with some sort of meaning. The fact that we already have started to treat *x-bots* as if they are human might lead to significant changes to our perception and practice of (adequate) social recognition, particularly as it relates to companionship. Given the different functional roles an *AIC* can play in a person's life, we might understand better why these objects mean so much to “their” humans: One marries the hologram *Hatsune Miku* (95)^6^, others share physical intimacy with love dolls (such as *Harmony*^7^)*,* some bury their AI pets, such as *Paro*^8^ or their love dolls in a proper farewell ceremony^9^, or do their work-out with *Pepper*^10^, use *Winky*^11^ for entertainment and pre-school education of children, or consult *Replika*^12^ for a romantic conversation. A lot of people find something (exclusively) in *x-bots* that either is evaluated as having some sort of additional value or beneficial effect for their lives, sometimes even because they can share with the *x-bot* something they would never address in relation to people. *AIC*s may even be experienced as preferable over humans with respect to performance qualities and it might be especially the artificiality—the “*as-if*”—that makes *AIC*s attractive to humans.

So, why not treat *AIC*s as being proper “(re-)productive sources” of social recognition? This still appears as puzzling, if we spare a second to remind ourselves how the sociability of humans fundamentally differs from “companionship” with a digital device: It seems that *AIC*s lack basically everything that is substantial to social recognition as it has been introduced here. The asymmetry that impregnates the human–*x-bot*s relation seems striking: (1) While human relatedness is characterized by the intersubjective dynamics of mutual social (re-)cognition, even the most “sociable” designed AI companion to date lacks *intersubjectivity* (albeit it normally is capable of some kind of inter-*action* and might even be ascribed self-referentiality). There is simply no “subject” that then would be capable for 291 a vital “*inter* –” beyond the mere “-activity” in the sense of a mutual recognition relation. It therefore still seems reasonable not to ascribe robots *consciousness* [cf. (96)], or an intentional self-relation [cf. (97)]. This alone seems the knock-out criterion for perceiving AI cognition in any form as equal, or adequate, for substituting human social (re)cognition practice. Nota bene: with this it is not said that they cannot minimally contribute to social recognition relations (which I believe is possible in some cases and is sketched in what follows). Quite to the contrary, others speak of AI “consciousness” in much more than a metaphorical way and draw strong analogies between the human consciousness and, for instance, the algorithmic activities of social AI (98–100). (2) Human praxis is the inter-*affective* praxis of sentient beings, which is dialogic in nature and encompasses all forms of (e.g., symbolic, physical, etc.) exchange, while an *AIC* simply traces and tracks emotional expressions, and/or mimics or triggers our emotions without having any sentient capacity. However impressive the recent developments of *affective computing* (101) and *responsiveness* 102, 103) might be, a robot capable of *caring*^13^ cannot be assumed (yet), even if these devices actually “do” care in a technical sense (104) and are therefore of instrumental value [cf. (105)] in particular fields of application [for questions of robot liability in care practice, see Beck et al. (106)]. (3) *AIC*s are (inter)active machines, but not *organically* (vital) social entities. Although *AIC*s, as forms of embodied cognition, possess “striving” and even spontaneity—due to the respective functional design of a binary code—there is no such thing as a *conatus* or freedom involved (which, when taken from one, elicits an essential suffering). (4) Some would even say that *AICs* neither have autonomy, nor any sort of agency that would come near to that of *self-reflexive* beings. An *AIC* is autonomous to run on its program if it has an engine, while persons need more than energy, namely, liberty for their autonomous self-actualization. This is the reason that social AI consequently can be considered *a-moral*, even if the design includes some kind of compatibility with ethical standards and implies rule following, according to which some perceive of *AIC*s as moral agents [cf. (107)]. Capurro (108) reminds that it is a dilution of the concept of morality if we assume that just because any agent can cause some good or its opposite that this necessarily implies some sort of moral accountability. Indeed, normally (if not otherwise intended by design) it is guaranteed that certain harm-norms are not transgressed by the *AIC* itself. Exceptions to this rule might be *x-bots* that could also be instrumentalized for harming (109, 110), or specific malware, which, for instance, can turn the chatbot Alexa into a “*Malexa*” (111). And finally, it can be doubted that mere pattern recognition, in a technical sense, is equivalent to that kind of *ethical* dimension that impregnates the term of social recognition that I have stressed here.

Nota bene: There are many ways of conceptually “dragging the soul into the machine” by mere definition. Normally this is done by using such criteria or specific readings of “capabilities” that then become re-conceptualized as structural relata to human “capacities” or a criterial definition of personhood, to *humanize AIC* in general, or to let them appear as somehow sufficiently capable of social recognition, in particular, because of a “match” or some strong conceptual analogies. I think this debate is basically (still) a matter of “belief” as robot consciousness is not (yet, to my knowledge) evidencable. I therefore suggest adopting a *pragmatic view* and counting in also the pro-arguments for *AIC*s as potentially contributing to the experience of social recognition: It could be simply accepted that human–object relations include libidinous investments and emotional attachment (112) to non-vital objects, too. The responses of *AIC*s (think of highly advanced social *x-bot*s, such as *Sophia*^14^) could probably be subjectively experienced and judged as “adequate,” particularly when the device contributes to achieving a particular human good; for instance: when it somehow beneficially offers someone attention or helps to prevent harm. If people can experience themselves as adequately recognized (i.e., loved, respected, truly seen, supported, desired, etc.) by a robot, or *trust* them (113, 114), we might have reason to consider a further extension of the sphere of recognition relations that somehow integrates this influence of *AICs* in loneliness treatment and prevention. We might not deal with an *inter-affective* mode of relatedness, but the possibilities that *x-bots* offer, in principle, simply cannot be ignored regarding loneliness management in the future. To give an example: The pathogen effects of social isolation in combination with a genetic vulnerability triggers the onset of schizophrenia and paranoia, as it has been exemplarily described in the case of a *lack of contact-paranoia* (115), especially in older people. So even the possibility of having a conversation or feeling that “some-*thing*” is around, might allow chronic loners to train their communicative skills, which could be helpful in preventing the onset or further development of the pathodynamics of loneliness. Such minimal contributions of *AIC*s could be valued; however, AI companionship that cannot be used as a medium to connect to other fellow beings—to my opinion—is still *monologic* in nature: we are actually (still) talking to ourselves! I have elsewhere called this the *echo chamber scenario* of AI companionship (24) that reveals the basic dilemma: there might be some sort of beneficial effect (the surface phenomenon of loneliness might be “eased”), but this remains potentially problematic, as the very basic condition that should be ideally altered is simply *reproduced*: we are connected, but alone! And even if certain criteria can be ascribed to *AIC*s in analogy to human capacities, I would (to date) still rule out intersubjective capacities in AICs, which I see—particularly with respect to the importance of inter-affective resonance and self-reflexivity for humans—as the basis for true recognition relations. I believe this is exactly what lonely people need to break free from their loneliness, but I would also accept that there are some people that perceive their exchange with an *AIC* as so fulfilling that they no longer *feel* alone. Another point is whether to assess this as a case of proper social integration, which I do not believe is necessarily given, even in scenarios in which someone is just fine with an *AIC*. It seems quite ironic to try to “fix” suffering from an altered relatedness to fellow-beings with a tool that in the best case just *appears* as human. The AIC–human relatedness is Janus-faced: one can certainly emphasize the instrumental value of social AI, but still must problematize the potential detrimental effects of *AIC*s, at least if the interventionist ideal would be that people really “break free from loneliness.” But what does this mean, and are there probable social AI scenarios that might be preferable to the type of (one-to-one) human–*x-bot* relation stressed so far?

### 2.3 How to leave loneliness?

We are intrigued by the *illusion of social recognition* as the *AIC* (un)cannily “simulates” affective attunement and understanding, and is often perceived as non-judgmental and accepting, which might be a balm to the wounded soul of people who feel neglected, misjudged, unappreciated by others, etc. This exact “comfort” might be a problem of *AIC*–human relationships, as on the surface some individual symptoms are “eased,” but people are still “objectively” lonely. By contrast, one might see the opportunity to enhance one's situation in relation to the *AIC* even if we are dealing with a “fake” recognition scenario. Coping with one's loneliness all alone might even be seen as an authentic expression of (digital) autonomy as this could demonstrate that someone is “in control” of their loneliness. From a psychological perspective, however, we must then be carefully reminded that autonomous enaction of individuals is always intertwined with the material reality of intersubjective practice, and that this is the decisive realm of agent autonomy. So, it can be assumed that as long as the psychic reality of a person stays connected to the material *reality* and their horizon of reasoning with other people, being attached to an *AIC* does not impair agency, although there is always a potential to “fall for the machine”, inasmuch as *AIC*s can be very appealing in showing “unconditional” appreciation and uncritical affirmation, which is constantly available to the user. Some transformative possibilities that may come with loneliness as an existentially challenging experience might be restricted for those who have an especially strong attachment to their *beloved object* (116) and may relapse into a “forgetfulness of social recognition,” i.e., of how important relations to fellow-beings are, even if this includes unpleasant experiences.

AI solutions come with different mediating and transformative potential that enable loners to get in contact with *real people*. I have claimed elsewhere (24) that *x-bots* should be designed in the future to serve this *transitional* function, i.e., helping to facilitate the transition from a position of social exclusion toward an active positioning of oneself (as a loner) in relation to others. Similarly to how one wears a cast for a few weeks to heal a broken bone, the use of an *AIC* could be restricted to prevent getting trapped within digital loneliness, i.e., a “fake” recognition scenario. A good example for AI as a medium to “leave loneliness behind” is the location-based reality game *Pokémon Go*, which provides a *digital community* with real road maps to go outside and hunt Pokémon. It has been shown that even chronic loners who have drastically socially withdrawn even for several years have become motivated to leave their digital loneliness to go outside and play with others [cf. (117, 118)]. Virtual reality spaces can motivate people to seek out relatedness with others and effective coping with loneliness seems to be much more likely when the opportunity to meet *fellow-beings* is given. This has been demonstrated in an Avatar Mediated Conversation setting (119), which users have evaluated as supportive in thematizing sensitive topics, such as their own loneliness experiences. These examples might not be the ultimate guarantee that one finally overcomes loneliness, but they offer much more possibilities for it to happen than being alone with an *AIC*. Of course, this depends again on the quality of the digital relationships; hence, it is crucial that symptomatic patterns of distorted recognition are not simply repeated in the virtual space. This will now be sketched to finally stress the critical potential of digital loneliness:

## 3 The critical potential of loneliness—the new precariat

The idea of loneliness as a *sociopathological* condition is compatible with the *sociological* and *culture-analytic* views on loneliness as mirroring a “defect in social relations” [cf. (120)] that cannot be combated with mere *digital connectedness* (121), and which retrospectively has impregnated the idea of loneliness as an “*inner homelessness*” (122) or “*mental isolation*” for which a “lack of individual ties and interpersonal shared values,” in particular, are essential (123). An active withdrawal from society to *voluntarily* isolate—or better: to distinguish oneself from others [e.g., as it is essential for Friedrich Nietzsche's loneliness concept, e.g., Nietzsche ZA1883-5, III (124)]—would allow one to perceive loneliness as a “*heroic*” mode of self-appropriation. However, these are very exceptional cases of assessing loneliness as a “valuable” form of social distancing.

Loners often must deal with not receiving even the most fundamental forms of social recognition, and therefore, often also withdraw to a kind of digital parallel universe (the “echo chamber” of AI): This is, for instance, the case with the so-called *hikikomori* (jap. 引きこもり) in Japan. The psychologist Tamaki (125), who coined the term, refers to this mode of loneliness as an abnormal avoidance of social contact that literally translates as “being confined.” Initial findings before the year 2000 showed that mostly young people were affected, while recently there has been a marked increase in *hikikomori* among the middle-aged and elderly people living in extreme levels of isolation, staying in the same room for a period of at least 6 months and refusing to leave the house. The digital contact to the outer world is maintained, but one's overall living situation is perceived as unsatisfying and depressing (126), in extreme examples resulting in the phenomenon of *kodoku-shi* (jap. 孤独死) (118), i.e., the solitary death. Apparently, the pressure that comes with societally up-held ideals, which are already impregnated by “system imperatives” (e.g., ideas of constant growth, development, and what counts as success in life, etc.) can lead people to completely withdraw from society and to become loners. Considering the classic alienation paradigm of *Critical Theory*, the digitalization of social companionship then is a systemically induced change of the lifeworld, in which system imperatives (“the digital agenda”) lead to an inner colonialization (127) of the sphere of the lifeworld, thereby potentially destabilizing important dynamics of social integration processes; mostly when economic imperatives infiltrate all eras of the public sphere, which then shows in the social miseries that are based on acceleration, reification, commodification, and therefore can be counted as expressions of alienation [see also Fraser et al. (128)]. Certain success goals whose significance and attraction are symbolically universalized within a cultural we-group may be prone to particular (misleading) readings of ideals, which has been exemplarily described by Ehrenberg (129) as a cause for chronic “exhausted societies” in which individuals suffer from depression and burnout. The same dynamics could also be the reason for social isolation and the increase of loneliness. If certain ideals that predetermine the success goals one “has to reach” [think e.g., of “perfectionism” (*kodawari;* jap. こだわり) or continual improvement (*kaizen*; jap. 改善), which are highly ranked norms not only in Japan] are perceived by a growing number of (younger) people as something that cannot be obtained or achieved, this most likely fosters tendencies of social disintegration, exclusion, and stigmatization, which can lead straight to loneliness. Merton (130) specified such criteria for success as causal factors for *anomistic* tendencies in societies. He particularly highlights the role of societal narratives, which often do not sufficiently communicate the fact that certain success goals and the means to achieve them simply cannot be acquired and reached by everyone, given the systemically induced inequality structures and limited resources. While some people actively socially withdraw from the pressure of success goals, others become chronically lonely according to different recognition struggles that come with age, or physical and mental impairments. What unites these groups is the struggle of being recognized as authorized recipients of certain forms of (societal) support (by family, peers, certain organizations, or governmental agencies). In addition to the monetary costs that loneliness causes (e.g., for the psychosocial healthcare sector) there is probably a much higher debt to pay with respect to the role that loneliness plays as a symptom of restricted social participation: the dynamics of *collective re-politization*, for instance, in the way of *radicalization*, must also be addressed alongside the notion of digital loneliness. This “discontent” with the analog world *and/or* within certain digital cultures—the *critical potential of loneliness*—is revealed in aggression. An example is the explicit misogyny in the Incel-community in which a gross majority of people (predominantly identified as male) are lonely, and thematize their experience of a lack of desired relatedness (131). This is just one among many other forms of (cultural) psychological reaction formations that can foster the social dynamics of ostracizing others, fanaticism, pathological (group) hate, and violence for which “digital loneliness” is the point of departure. This is the dark side of loneliness: the envy, jealousness, hatred, or revenge fantasies, which Freud (132) would have grouped as aggressive relapses under the diagnostic category of *culture hostility* (dt. *Kulturfeindseligkeit*). They are directed by “unrecognized loners” particularly toward those groups or individuals that supposedly receive all the precious recognition—and the associated resources—that one would like to have for oneself. This is the negative counterpart to a silent (digital) suffering from loneliness and poses another important ethical problem—besides the risk that loners might get trapped in fake recognition scenarios with social AI—that must critically be assessed by addressing distorted recognition as an institutionalized practice for intersubjective exchange in certain (digital) niches.

It can be concluded that loneliness inevitably becomes socially visible, particularly the digital realms in which it can “flourish”—may it be in terms of resentment or in terms of depression and hopelessness about the very condition of feeling and being socially disadvantaged (133). If loneliness leads to anomic states that are intersubjectively consensually evaluable as harmful or distressing not only for those directly affected, but also for others, societies with growing rates of loneliness should be concerned about whether social AI, particularly *AIC*s, really are sufficient tools to deal with these socially precarious dynamics. Despite the enthusiasm about the future possibilities that will come with further humanizing AI, a critical (not pessimistic) view on AI companionship must accompany it if there is something correct about my claim that social AI plays a causally explanatory role for the growing problem of and the specific non-trivially harmful forms of loneliness in recent societies. The idea that digital connectedness automatically positively alters precarious situations of loneliness is dismantled as a myth. In the future, the *x-bot*, the *AIC*—the artifact that is treated as if it is human—will continue to uncannily intrude into our lifeworld in which loneliness counts as *the* new precariat. This points to the risks of the AI agenda: The very aim of trying to solve loneliness with AI *cognition* appears misled with respect to the duties that come with the ethics of social *re*cognition.

## 4 Conclusion

The aim of this analysis was to provide some reason to accept that the lifeworldly logics of recognition cannot be easily substituted by AI cognition. I have adopted a culture-reflexive, critical view on digital loneliness (management). If loneliness is basically a (collective) cry for the need for social recognition, it seems that (to date) AI cognition cannot replace, substitute, or compensate for human recognition in such a way that makes it ethically justifiable to further outsource the (re-)production conditions of social integration to AI. Loneliness refers to the state in which one is connected to others or related to an *AIC*, but still is deprived of meaningful (i.e., a specific qualitative) relatedness in terms of empathy, mutual respect, und understanding. In those digital scenarios where this is actually experienced with an *x-bot*, one can pragmatically accept that there are cases in which *AIC*s might offer social recognition to people, although it has simultaneously become stressed that this might be the point of departure for the manifestation of chronic loneliness, which must be further determined by empirical research.

Understanding loneliness requires us to ethically tackle both the risk of becoming even lonelier in a monologue staged by a machine that is designed to let us forget about its echo chamber nature, and the risk of the reproduction of loneliness in being exposed to “active forgetfulness” by our fellow beings. To ensure it does not become a cultural trap, but a possible cure, what I suggest predominantly matters is not how advanced the *x-robots* are, but how advanced a society is in tracking and preventing deficiencies or false recognition as structural deficits that cause social maladies such as loneliness. This critique of established social narratives on “social” AI points out that well-digitalized societies should invest in humane institutions in the first place to create adequate ethical embedding conditions for any further institutionalization of humanoids.

## Footnotes

^1^The term “social x-bots” “can be used to refer to all types of digital “companions” (hereafter also referred to as *AIC*s) that can be integrated into or are designed to adapt to human relational dynamics (e.g., specific communicative settings) and show differences in terms of the specificity of embodiment, interface capabilities, and system coupling. I am aware that there are plenty of different types of robots such as *Winky*, *Aibo*, *MiRo, Paro, EmotiRob, Pepper, Dinsow, ElliQ, Atlas, Asimo, Harmony, LOVOT,* or conversational agents or chatbots such as *ELIZA*, *Alexa*, *XiaoIc*e, *Replika*, *Tess, Woebot*, and *Wysa*, etc. Relevant for this analysis is that all these *x-bots* create *illusions* of intersubjective/inter-affective exchange, but might still not be seen as adequate to solve the problem of a lack of meaningful relatedness in loneliness.

^2^Japan’s annual birth rate fell below 1 million in 2016 for the first time since 1899, leading to a boom of social AI in the form of care-assisting robots for dealing with the high gerontification. According to data from the *Ministry of Internal Affairs and Communications*, the number of people over 65 has risen to 36.4 million. Japan's *Ministry of Economy, Trade and Industry* forecasts that the robot service industry will grow to nearly $4 billion annually by 2035, which is 25 times its current value (09/19/2021stat.go.jp; accessed July 28, 2023).

^3^*Non-trivial* refers to suffering from loneliness in a (social) pathological relevant sense as distinct from other forms of suffering from it that do not imply the biopsychosocial malfunctioning associated with loneliness as a persistent (“chronic”) condition.

^4^The *Demographic Change and Healthy Ageing Unit* of the WHO has announced the UN Decade of Healthy Ageing (2021–2030) and addresses social isolation and loneliness as one of the most pressing topics of health promotion, particularly for the elderly population also under the auspices of digital interventions (such as skills training, community and support groups, and cognitive behavioral therapy) that have been developed to reduce social isolation among older people, with the aim of improving access to information and communication technologies in order to create a more age friendly community, which comes with an interventionist claim that seem to have been overlooked for a long time.

^5^Language […] has created the word “loneliness” to express the pain of being alone. [A]nd it has created the word “solitude” to the glory of being alone (Tillich 1963, p. 17).

^6^The Japanese mangaka and illustrator Kei Garo *Miku Hatsune* designed the virtual character *Hatsune Miku* on behalf of Crypton Future Media, and the company *Gatebox* uses this character for their companion chatbot. Akihiko Kondo officially married the virtual figure Hatsune Miku. See https://www.otaquest.com/hatsune-miku-gatebox-marriage/ (accessed November 25, 2022).

^7^*Harmony* is a RealDoll companion robot that allows customers to create their doll by choosing among 10 “persona points” to customize it according to individual preferences. https://www.althumans.com/companion-robots/real-doll.html (accessed August 17, 2023).

^8^*Paro* is an AI pet that resembles a Canadian Harp seal pup. It has been in use in nursing facilities in Japan since 2003. It responds to tactile stimuli and recognizes temperature, posture, and light.

^9^Leiya Arata is a founder of the *Love Doll Funeral* services in Osaka. For $800 customers can have a mannequin memorial. Presiding over the mannequin memorials is the Buddhist monk Lay Kato. There is also fare-well ceremony for AI pets: When Sony announced in 2014 that they would no longer support updates for AIBO, the community of owners began sharing tips on providing care for their digital friends in the absence of official support.

^10^*Pepper* is called a “semi-humanoid” robot manufactured by SoftBank Robotics (formerly Aldebaran Robotics). Its crucial feature is emotion detection based on face and voice tone analysis. Pepper simulates active listening with arm and hand gestures, which creates the appearance of having self-awareness. It was introduced in June 2014 in Japan.

^11^*Winky* is a play-bot produced by the company *Mainbot* and comes equipped with a microphone, sensors, a speaker, LEDs, a rotating head and ears, a motion and distance detector, and a gyroscope for interacting with the environment.

^12^*Replika* is a generative AI chatbot that was released to the public in 2017 and within 1 year was used by 2 million people. The user must answer a series of questions to create a network that serves as contextual frame for the “friendship”—including romantic and, before the function was disabled by the developers in 2023, erotic relationships.

^13^“Caring” is not a solely morally neutral concept but is bound to ideas about the good life. To count as caring, the design of *x-bot*s would have to include a reflexive self-relation which manifests itself in acts of caring *as* moral self-realization. Even if an *AIC* is responding to its object of care, there is no “knowing” as it has no idea of its own wellbeing or the wellbeing of others. But it is exactly this that qualifies caring as the mode of being a moral person.

^14^Hanson Robotics has developed *Sophia*. In 2017, it was given citizenship and was the first non-human to be given a United Nations title (UND Programme's first Innovation Champion).
